# Auditory Stimulation Training With Technically Manipulated Musical Material in Preschool Children With Specific Language Impairments: An Explorative Study

**DOI:** 10.3389/fpsyg.2019.02026

**Published:** 2019-09-04

**Authors:** Ingo Roden, Kaija Früchtenicht, Gunter Kreutz, Friedrich Linderkamp, Dietmar Grube

**Affiliations:** ^1^Department of Educational Psychology, Carl von Ossietzky University of Oldenburg, Oldenburg, Germany; ^2^Speech and Music Lab, Carl von Ossietzky University of Oldenburg, Oldenburg, Germany; ^3^School of Education, University of Wuppertal, Wuppertal, Germany

**Keywords:** auditory stimulation, working memory, language disorders, transfer (psychology), preschool children

## Abstract

Auditory stimulation training (AST) has been proposed as a potential treatment for children with specific language impairments (SLI). The current study was designed to test this assumption by using an AST with technically modulated musical material (ASTM) in a randomized control group design. A total of 101 preschool children (62 male, 39 females; mean age = 4.52 years, SD = 0.62) with deficits in speech comprehension and poor working memory capacity were randomly allocated into one of two treatment groups or a control group. Children in the ASTM group (*n* = 40) received three 30-min sessions per week over 12 weeks, whereas children in the comparison group received pedagogical activities during these intervals (*n* = 24). Children in the control group (*n* = 37) received no treatment. Working memory, phoneme discrimination and speech perception skills were tested prior to (baseline) and after treatment. Children in the ASTM group showed significantly greater working memory capacity, speech perception, and phoneme discrimination skills after treatment, whereas children in the other groups did not show such improvement. Taken together, these results suggest that ASTM can enhance auditory cognitive performance in children with SLI.

## Introduction

The National Institute of Deafness and Other Communicative Diseases (NIDCD) reported that 7 to 10% of 5-year-old children are diagnosed with specific language impairment (SLI). Typically, these children have problems with specific rules of grammar, such as the misuse of verb tense (Reilly et al., 2014). There appears to be consensus that SLI originates from deficits in low-level auditory processing of both linguistic and other sound materials (Fey et al., 2011; Murphy and Schochat, 2013). This hypothesis has led to the development of so-called auditory stimulation training (AST) to address children with SLI. However, identifying key elements and evaluating them across different AST programs has remained a daunting task. Here, we explore the efficiency of modulated musical sound materials as produced by a commercially available system (AUDIVA®) to stimulate auditory processing in children with SLI. Specifically, we addressed how this training would affect the cognitive processing of linguistic materials and auditory working memory in this target group.

### Auditory Stimulation Training and Language Skills

Murphy and Schochat (2013) conducted a systematic review and found a total of 29 studies eligible for review, which were categorized in terms of three different types of AST: software-based training, formal auditory temporal training, and music training. The first type of AST representing a majority of these studies entailed software programs including FastForWord, Earobics, AudioTraining or STAR (Cognitive Concepts, 1997; Scientific Learning Corporation, 1998). Briefly, these software programs represent different types of tasks that are related to linguistic processing (e.g., listen-and-repeat tasks) and, in few cases, cognitive tasks (e.g., working memory training). Some of these programs manipulate the rate and intensity of speech components of frequently repeated linguistic and non-linguistic stimuli and/or manipulate language components, such as vowels, consonants and syllables to improve language processing. Auditory and language learning gains are indicated in these studies with respect to behavioral (Tallal et al., 1996; Strehlow et al., 2006; Fisher et al., 2009; Murphy and Schochat, 2013) and electrophysiological measures (Hayes et al., 2003; Russo et al., 2005; Heim et al., 2013). These findings support the hypothesis that holds that AST training leads to significant increases in language outcomes and enhanced auditory temporal processing. However, Murphy and Schochat (2013) identified risks of biases in some of the studies, which limit the interpretation of their findings.

Two of the studies cited above focused on children with SLI; thus, they appear most relevant to the present endeavor (Tallal et al., 1996; Heim et al., 2013). Tallal et al. (1996) tested the assumption that acoustically modified speech with rapidly changing elements in the acoustic waveform should facilitate acoustic processing in children with SLI. The authors indeed found significantly improved speech discrimination and language comprehension abilities, albeit there was no control or comparison group to substantiate this interpretation. Heim et al. (2013) examined whether the temporal precision of neural coding in children with SLI might be improved via intensive, software-based audio-visual training. The learners attend visually presented exercises that are accompanied by increasingly complex acoustic events such as frequency sweeps, phonemes, words, and sentences. After 32 days of training, the authors observed behavioral improvements in measures of language comprehension. They also found that children improved their temporal auditory processing skills via AST. However, the absence of an alternative training group compromises this interpretation. In other words, whether the observed effects were specifically related to this type of AST remains to be demonstrated.

The second type of studies according to Murphy and Schochat (2013) evaluated formal auditory temporal training in an acoustic cabin. Researchers applied similar tasks including auditory closure, temporal ordering, sentence and non-verbal sounds or figure to ground for digits tasks (Miranda et al., 2008; Gil and Iorio, 2010; Megale et al., 2010; Schochat et al., 2010; Vilela et al., 2012). However, only one study addressed 7- to 12-year-old children with language disorders; children with SLI were not targeted (Filippini et al., 2012). This study suggested improved auditory temporal skills in these children after formal auditory training, whereas no such improvements were found in either typically developed children or children with language disorders.

Fey et al. (2011) explored the efficacy of auditory and language interventions for school-aged children with auditory processing disorders in an evidence-based systematic review. The authors reviewed eleven studies that examined the effects of auditory interventions on language outcomes for children with spoken language disorders. A majority of these articles evaluated the effects of Fast ForWord or Fast ForWord-like interventions. Six of eleven studies were classified as exploratory research. Most of the studies reported overall positive outcomes, while two studies found high improvements on standardized measures of receptive language (e.g., Tallal et al., 1996; Stevens et al., 2008).

Some studies using the Fast ForWord program, however, produced limited or no significant gains (e.g., Loeb et al., 2001; Agnew et al., 2004). Interestingly, significant decreases in receptive language skills were also noted (e.g., Friel-Patti et al., 2001; Loeb et al., 2001).

Moreover, Fey et al. (2011) reported different outcomes for five efficacy studies and two language-oriented auditory interventions. On the one hand, studies by Alexander and Frost (1982), Tallal et al. (1996), Gillam et al. (2001), and Cohen et al. (2005) showed the benefits of standardized measures of phonological awareness, grammar comprehension and language tests. However, those benefits were only reported for a few secondary measures (Gillam et al., 2001; Cohen et al., 2005), which calls into question the efficacy of the interventions. Pokorni et al. (2004) examined different types of interventions, such as Fast ForWord, LiPS (Lindamood Bell Learning Processes, 1999) and EROBICS (Cognitive Concepts, 1996). The authors did not report any group differences for phonological awareness or language outcomes, besides the Blending Phonemes subtest, in which children with auditory stimulation training based on a phoneme sequencing computer program (LiPS) showed greater improvements than their controls. Finally, the two language-oriented auditory interventions by Bishop et al. (2006) and (Segers and Verhoeven, 2004) did not show any significant gains in auditory processing or language tests or in phonological awareness tasks. In summary, Fey et al. (2011) suggested that the effects of AST for children with language disorders had not been examined adequately.

Other approaches focused on computer-based multisensory learning programs in which the memory of phonemes and graphemes should be strengthened by visual and auditory associations to improve both reading and spelling skills in children with and without dyslexia (Kujala et al., 2001; Kast et al., 2007, 2011; Ecalle et al., 2009). For example, a study by Kast et al. (2007) used a spelling software called “Dybuster” that recodes words into multisensory representations with visual and auditory codes, to alleviate writing errors in children with and without dyslexia. Results from a matched control group design with pre-post measurement showed that children with dyslexia strongly improved their writing skills after 3-month of visual-auditory multimedia training compared to children with dyslexia who do not received a training. Interestingly, children with dyslexia who received the training also improved their writing performances for words that were not part of the training software, indicating a strong transfer effect of the training. Even children without impaired writing or reading skills improved theirs writing skills through the training (Kast et al., 2007).

Taken together, AST appears to be a promising route in the rehabilitation of children with SLI. Nevertheless, the effectiveness of relatively new approaches to AST such as the use of software tools for speech manipulation, language-oriented auditory interventions or multisensory learning programs is still an ongoing debate (see Kujala et al., 2001; Kast et al., 2007; Fey et al., 2011; Kast et al., 2011; Murphy and Schochat, 2013). Moreover, the different types of AST and the small number of studies make it difficult to determine the efficacy of a particular intervention technique and its general benefits for language skills for children with SLI.

### Auditory Stimulation Training and Music

Apart from those results from AST in speech rehabilitation, the effects of music on language-related skills are difficult to compare, and evaluating their overall efficiency is difficult. For example, Kampfe et al. (2010) examined the effects of background music on reading skills, whereas the systematic review of Murphy and Schochat (2013) mainly focused on studies using acoustically modified speech or music processing algorithms. Other approaches examined the impact of music training on language and early literacy skills. Those studies were based on different types of musical interventions, such as playing a musical instrument or singing, and included a wide scope of participants, ranging from infants to typically developed children over children with dyslexia and cochlear implants (Dege and Schwarzer, 2011; Francois et al., 2013; Fonseca-Mora et al., 2015; Gordon et al., 2015; Wiens and Gordon, 2018; Steinbrink et al., 2019). In summary, those studies provide consistent support for the inclusion of music instruction in early childhood (Gordon et al., 2015; Wiens and Gordon, 2018).

Although there is an association between musical behaviors and cognitive performance with respect to auditory processing skills in different domains, it is less clear whether and how this association extends to specific forms of music instruction and to children with language comprehension deficits (see Murphy and Schochat, 2013). Despite the absence of studies focusing on the use of auditory stimulation training with technically modulated musical material (ASTM) to improve language outcomes, the approach is based on studies that were published in the 1970s and 1990s (Tallal and Piercy, 1973; Fitch et al., 1997). For example, Tallal and Piercy (1973) proposed that SLI might be related to deficits in auditory temporal processing. Further research in the 1980s and 1990s supported this hypothesis (e.g., Tallal, 1980; Fitch et al., 1997). In particular, Habib (2000) observed difficulties in the processing of temporal auditory stimuli when the stimuli were presented in rapid succession (Habib, 2000). The author suggested that a limited capacity to process short acoustical information such as vowels and consonants can lead to difficulties in the association of letters and their specific sounds. This lack of encoding could then compromise the sensory-motor mapping of letters and their conversion to phoneme production. Moreover, Steinbrink et al. (2014) concluded from their cohort study that auditory processing abilities causally influenced individual literacy and language development. Further indirect support for the use of ASTM could be found in studies focusing on the speech rhythm in specific language-impaired or dyslexic children across languages. For example, an investigation by Cumming et al. (2015) showed a significant relationship between auditory processing skills and sensitivity to rhythmic timing, which is also a key aspect of music perception and processing (see Goswami, 2010; Huss et al., 2011; Tierney and Kraus, 2013; Woodruff Carr and White-Schwoch, 2014).

Up to this point, strategies to improve auditory speech comprehension at psychoacoustic levels are considered key to facilitate language encoding (e.g., Goh and Taib, 2006). As rhythmic and tonal information is explicit in music, auditory stimulation with musical material might have beneficial effects on the process of auditory perception in children with SLI and dyslexia. One of the first studies to investigate the effects of music listening in children with dyslexia was from Overy and colleagues (Overy, 2000; Overy et al., 2003). They tested 15 dyslexic and 11 typically language-developed children with a specifically designed collection of musical aptitude tests, which made it possible to distinguish among a variety of musical skills (mainly pitch and rhythm). The results showed that children with dyslexia scored higher on pitch skills and lower on rhythm (timing) skills than their counterparts. In addition, Overy et al. (2003) reported a strong correlation between rhythm tapping and spelling abilities, suggesting that the impairment of phonological abilities for children with SLI is linked to the general deficit in processing timing information. Due to the quasi-experimental design, the relatively small sample size, and the non-matched controls, the results must be treated with caution. However, a study by Flaugnacco et al. (2014) support these findings, by examining rhythm perception and production skills as predictors for reading abilities in children with dyslexia.

### Rhythm in Music and Language

In the past decade, there were a growing number of investigations into the idea that rhythmic skills in particular might be important for the development of language and literacy skills in typically developed children and children with language disorders, including SLI and dyslexia (e.g., Goswami, 2010; Huss et al., 2011; Tierney and Kraus, 2013; Woodruff Carr and White-Schwoch, 2014). Furthermore, evidence has increasingly shown that children with SLI and dyslexia show impaired temporal processing in both language and music (Corriveau et al., 2007; Brandt et al., 2012; Flaugnacco et al., 2014; Clément et al., 2015; Planchou et al., 2015). In particular, children with dyslexia show deficits in perceiving tempo information and tapping to a default rhythm (Thomson and Goswami, 2008; Corriveau and Goswami, 2009). Sallat and Jentschke (2015) examined the melodic and rhythmic-melodic perception of short musical pieces in 5-year-old children with SLI and two control groups. Controls included children with typical language development (TLD) of the same age as the intervention group and a group of younger children (4 years old) with language skills comparable to those of the children with SLI. The authors hypothesized that the processing of prosodic information involves skills similar to those required in music perception. The results showed that children in the SLI group performed equally to the younger children and poorly compared with the children with TLD. Along these lines, the authors suggested the use of musical material in therapy for children with SLI. This interpretation is consistent with previous research reporting that rhythmic auditory stimulation has the potential to boost linguistic structure processing (Hannon and Johnson, 2005; Przybylski et al., 2013; Cumming et al., 2015; Planchou et al., 2015).

### Auditory Stimulation Training With Music for Children With SLI

Despite anecdotal reports of positive effects of ASTM in children with SLI, there are no systematic reports available that address these issues in studies with a controlled longitudinal experimental design. Moreover, none of the reported studies focused on the effects of ASTM on auditory working memory performance. However, working memory lends itself as a cognitive system to be affected not only by music training but also by music listening. As a mental system responsible for temporary storage and simultaneous manipulation of information, it is involved in many kinds of conscious mental processes, such as auditory processing and language comprehension (Baddeley, 2006; Franssen et al., 2006; Roden et al., 2012). Hence, an ASTM that increases the perception and the procession of auditory information might also be helpful in developing efficient articulatory rehearsal strategies and positively affect auditory working memory capacity in children with SLI. Finally, none of the previous studies focused on an ASTM to improve language outcomes. Therefore, the aim of the present study was to explore the effects of ASTM in preschool children with SLI on auditory working memory, speech perception and phoneme discrimination performance. Because certain consonant sounds have primary frequencies above 3,000 Hz, the musical stimuli were manipulated as described. First, we only used high-frequency music by including classical pieces of Mozart, Bach and Vivaldi, which were rich in overtones. Second, the music stimuli were filtered to remove the low frequencies (<1,000 Hz) and boost the high frequencies (>2,000 Hz) of the music signal. Finally, sound delivery was lateralized such that frequencies presented to the left ear at higher levels were attenuated to the right ear and vice versa. Based on previous findings, this approach shows that the collaboration of both hemispheres of the brain is essential for phoneme detection and speech comprehension: the superior posterior part of the left temporal lobe (Broca’s area) and adjacent partial regions, located in the parietal and temporal lobe of the Wernicke’s area, and areas of the right hemisphere, which are responsible for speech perception and the production of prosodic qualities of speech (see Ryding et al., 1996; Ross et al., 1997). To clearly attribute the observed effects of our ASTM on the dependent variables, we designed an explorative randomized control group study with two time points of measurement. The study protocol also included screening procedures to assess language impairments and specific speech-comprehension deficits.

The present study aimed at investigating these tentative findings further by testing the following hypotheses. First, we hypothesized that children in the ASTM group would show a higher increase in auditory working memory performance after 12 weeks of intervention than would children in the control groups (H1). Second, we hypothesized that children in the ASTM group would outperform controls concerning their speech perception skills at high frequencies from 4,000 to 2,000 Hz (H2). Finally, we hypothesized that children in the ASTM group would show higher phoneme discrimination skills than controls would after the end of the intervention (H3).

## Materials and Methods

### Participants

A total of 141 children from 10 preschools located in rural areas of the state of Lower Saxony, Germany, were first selected based on deficits, as identified by interviewing their kindergarten teachers, in any of the following domains: poor listening and memory abilities, easily distractible attention, sensitivity to noise and language that is difficult to understand. Each child was then submitted to a test battery to assess their understanding of basic grammar rules (TROG-D; Von Fox-Boyer, 2009, German adaptation of TROG; Bishop, 1989) and to a standardized auditory screening test (HASE; Schöler and Brunner, 2009). Both tests were used to identify children with SLI. Only children with low percentile ranges for the *Test of Reception of Grammar* (TROG-D) and with poor performance on one or more subtests of the Auditory Screening Test (HASE), which were defined separately for monolingual and multilingual children in the manual, were further included in the study. In contrast, children with mental disorders, psychological trauma and psychoses, children who used hearing aids, children who permanently replaced the phoneme |k| and |g| with |t| or |d|, and children with an acute illness were excluded from the study. Moreover, children from bilingual families who needed to acquire two formal language systems, were also excluded from the study.

Based on these inclusion and exclusion criteria, a total of *N* = 101 preschool children (mean age = 4.52 years; *SD* = 0.59; 62 males, 39 females) from seven different kindergartens were randomly assigned to the AST group, the pedagogical activity control group, and the control group. A power analysis using G^∗^Power (Erdfelder et al., 1996) suggested that the sample size was sufficient to ascertain small to medium effects (*f* = 0.25) in a mixed within-/between-subject design (α:0.05, power (1-β):0.80, correlations between repeated measures: *r* = 0.50).

Both interventions (ASTM and PA) took place in kindergartens with different room sizes. Therefore, the allocation of the groups was not divided equally. Forty children (24 male, 16 female) participated in the auditory stimulation group with technically manipulated musical material (ASTM), and twenty-four children (16 male, 8 female) joined the pedagogical activities group (PA). Finally, a total of thirty-seven children (22 male, 15 female) served as waiting controls (CG). Children in the CG had the opportunity to participate in either the ASTM or the PA training after the intervention phase of the present study ended. However, no additional data were collected from these children. One-way between-groups analyses of variance (ANOVA) and chi^2^ tests were conducted to assess possible baseline differences between groups. In summary, there was no difference between groups for any independent (Age, *Test of Reception of Grammar;* all *F*_*s*_ < 1.99, *all p_*s*_* > 0.14*;* Gender: χ*^2^* = 0.37; *df* = 2; *p* = 0.83) or dependent variables (Digit Span, Non-word Recall, Recall of Sentences, *Phoneme Discrimination* and the *Speech Perception at high frequencies;* all *F_*s*_* < 1.33, *all p_*s*_* > 0.27) at baseline, indicating that the randomization successfully minimized or avoid systematic bias in the selection process. Migration background between groups was by 13% for each group.

### Measurement Instruments Test of Reception of Grammar (TROG-D)

The German adaptation of Bishop’s *Test of Reception of Grammar* (*TROG*; Bishop, 1989) by Von Fox-Boyer (2009) was administered to measure the verbal comprehension of syntax. Each of the 84 test stimuli was presented in a four-image multiple-choice format with lexical and grammatical foils. Three images were slightly different in terms of the grammar and lexicon of the respective target. For each of the 84 stimuli, a sentence is read to the child. The participants were asked to select one of four images to match the sentence. For example, the corresponding sentence is “The cats were looking at the ball.” Only one of four images will match the sentence. The other three images showed (a) two people playing with a ball, (b) two cats looking at a butterfly, and (c) one cat looking at a ball (see Supplementary Appendix 2 for the corresponding image).

Twenty-one grammatical phenomena were administered in test blocks of four spoken sentences with 16 different images. Only if all four sentences of a test block were answered correctly was the test block considered solved. The results were assessed by a total raw score of correctly solved test blocks. The *TROG-D* is standardized for children between 3.0 and 10.11 years of age. According to the manual, 4-year-old children with a percentile rank ≤41, 5-year-old children with a percentile rank ≤40, and 6-year-old children with a percentile rank ≤39 were categorized as children with specific language impairments (SLI). The *TROG-D* reports a high Cronbach’s alpha (α = 0.86) and split-half reliability measures (*r* = 0.87), representing good internal consistency.

#### Auditory Screening Test (HASE)

Assuming that an insufficient understanding of language may be related to limited auditory memory abilities (Vukovic and Siegel, 2006), a standardized auditory screening test (HASE; Schöler and Brunner, 2009) for preschool children was used for children who performed poorly on the TROG-D test. The screening provides information on language comprehension and production and on auditory working memory performance. Three of four subtests were used: the *Digit Span Test*, the *Non-word Recall Test*, and the *Recall of Sentences Test*. Each test includes standardized instructions for both administrators and participants. Cronbach’s alpha for the three subtests varied between α = 0.71 and α = 0.83.

##### Digit span test

The reproduction of number sequences (digit span) is considered a valid indicator of phonological loop capacity (Hasselhorn et al., 2012; Roden et al., 2014). Based on the fact that the number of syllables significantly influences auditory working memory performance, only monosyllabic numbers from zero to ten were used, excluding the number seven (Schöler and Brunner, 2009). During the task, several sequences of one-syllable numbers were aurally presented via headphones (2 × 2 numbers, 2 × 3 numbers, 2 × 4 numbers, etc.). The children were required to recall the numbers in the correct chronological order directly after hearing the last number of the sequence. If the recall was correct for two consecutive trials, the trial counted as two points. If only one trial of a sequence was correct, it counted for one point. If the recall failed for four trials in succession, the test ended. The maximum score was ten points, and the longest sequence of numbers was six.

##### Non-word recall test

The immediate recall of non-words is a measure of the phonological short term memory. In particular, the *Non-word Recall Test* measured how accurately a participant can store unfamiliar words through the articulatory rehearsal mechanism (Gathercole, 1996). The use of non-words reduces the potential influence of long-term memory to reconstruct the sound or phoneme structure of a perceived word. During the task, ten three- to five-syllable non-words (e.g., “lufa” or “fodekina”) were aurally presented via headphones (65 dB). The non-words systematically differed from each other in terms of vowel and consonant order and vowel position. After the presentation of each non-word, the participants were asked to reproduce the word as accurately as possible. Each correctly recalled non-word counted as one point. A maximum of ten points could be reached. No adaptive test design was applied here. Therefore, the test procedure was repeated after all ten non-words had been presented and recalled.

The *Recall of Sentence Test* measures the production and understanding of language expressions. It also yields a measure of the short-term processing of language. The complete test consists of ten pairs of sentences (a and b). Each pair consists of two different sentences with the same sentence structure (e.g., “The shirt gets ironed” or “The dog gets fed”). The second sentence was presented only if the recall of the first sentence was incorrect. The level of difficulty was systematically increased and varied from short sentences with simple structures (such as “Peter is running”) to longer sentences with more-complex syntactical structures (such as “The big lamp hangs over the table in the living room”). Each correctly recalled sentence (a or b) was counted as one point. The maximum task score was 10 points.

#### Phoneme Discrimination Test

The *Phoneme Discrimination Test* measures the selective discrimination of phonemes entailing similar consonants in the German language, such as b/d, d/t, g/k, and f/w. During the first task, children were asked to immediately recall 32 different two-syllable non-words (such as “AFI,” “IDA,” EBU”; for a full list of stimuli, see Supplementary Appendix 3) that were presented in alternation in the left and right ears via headphones. Each correctly recalled consonant of the presented non-word counted as one point, up to a maximum of 32 points. The test procedure continued after all 32 non-words had been presented and recalled. In addition, 32 different two-syllable non-words were presented with background noise, which impedes the correct identification of consonants. To adjust the noise to the hearing threshold level, the noise was filtered across a frequency range from 100 Hz to 20 KHz. Again, children were asked to recall the non-words immediately after hearing. Correlation between the two time points for the *Phoneme Discrimination Test without background noise* was *r* = 0.42 and was *r* = 0.31 for the *Phoneme Discrimination Test with background noise*.

#### Speech Perception at High Frequencies

The *Speech Perception Test* was another recall task in which a word list of 10 three-syllable words from basic vocabulary were presented (specifically, “Ein-kau-fen,” “Aus-trin-ken,” “Ein-pflan-zen,” “Auf-ste-hen,” “Hin-brin-gen,” “An-zie-hen,” “Umb-lät-tern,” “Auf-es-sen,” “Hin-set-zen,” and “An-schrau-ben”). Stimuli were filtered to remove frequencies below (a) 4,000 Hz, (b) 3,000 Hz, and (c) 2,000 Hz. In other words, the acoustic characteristics of the vowel formants of the phonemes were changed. The task was performed with descending difficulty levels from 4,000 to 2,000 Hz. Children were asked to immediately recall a word after hearing. Re-test reliabilities varied among *r_*tt*_* = 0.31 (for 2,000 HZ), *r_*tt*_* = 0.38 (for 3,000 Hz), and *r_*tt*_* = 0.60 (for 4,000 Hz).

### Intervention and Control Conditions

#### Auditory Stimulation Training With Technically Manipulated Musical Material (ASTM)

The preschool teachers and child care workers who conducted the ASTM were trained by research assistants. Data at both time points were administered by research assistants only. The commercial hardware system used for the ASTM is called “AUDIVA-HWT^1^”. It contains a Discman (AEG, CDP109), a headphone interface for five headphones (KV-2) and full-sized headphones (QP 160) with a transmission range from 30 to 26,000 Hz. The volume level at the hardware device was fixed at 65 dB (SPL). Because certain consonant sounds have primary frequencies above 3,000 Hz, the musical stimuli were manipulated as described below.

First, we only used high frequency music by including classical pieces of Mozart, Bach and Vivaldi, which were rich in overtones. In particular, the selected music pieces contained 14 different pieces from Mozart (Rondo Allegro KV 182, 216, 218, 314, Un poco adagio KV211a, Allegro aperto KV 314, Menuetto allegretto KV 525 and Violin Sonate KV 301), Bach (Allegromoderato BWV 1041, Allegro BWV 1042, Allegro assai BWV 1042 and Vivace BWV 1043) and Vivaldi (Presto Op3 and Allegro RV 540). The music pieces are fully orchestrated and performed with stringed, plucked, and woodwind instruments.

**FIGURE 1:**
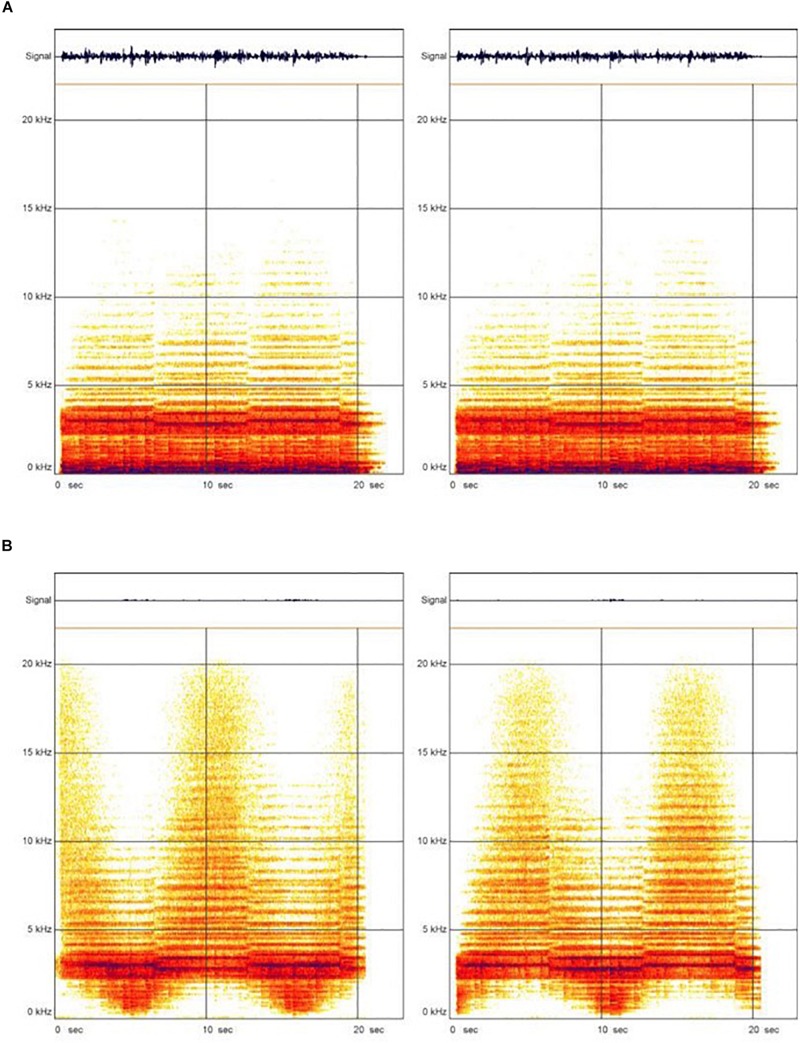
Visualization of the frequency spectrum of the ASTM. Condition **(A)** represents the visualization of the frequency spectrum of the ASTM with unmanipulated music. The dark area represented the sound volume of the orchestra, the brighter area were the solo instruments. The horizontal stripes that emerged were the natural overtones of the violins. Condition **(B)** showed the frequency spectrum with manipulated music, where high and medium frequencies (>2000 Hz) are compressed at intervals. Moreover, the medium and high frequencies were lateralized in a way that they were strongly condensed on the left ear while being shut down on the right ear at the same time and vice versa.

Second, the ASTM included electronic filters that removed low frequencies (<1,000 Hz) and boosted medium and high frequencies (>2,000 Hz) of the music signal to adjust its frequency levels to the primary frequency levels of speech comprehension (see Figure 1 for a visualization of the frequency spectrum of the ASTM with unmanipulated or manipulated musical material). Finally, the medium and high frequencies were lateralized such that they were strongly condensed on the left ear while being shut down on the right ear at the same time and vice versa. Thus, a lateral temporal change in an adjustable time (2–25 s) was given (see Supplementary Data Sheets 1, 2). The ASTM could be adjusted to six levels to determine both the frequency level of the filtering signal and the duration of the lateralization process in seconds. In a standard session of 30 min, a child heard acoustically modified music over earphones in a small group of 5–6 children. During the intervention phase, the frequency level and the duration of the lateralization process both increased from level 1 to level 6 to avoid habituation effects in the hearing process. At level 1 and level 2, lower frequencies (0 to 1 kHz) were separated from the tonic tones (3,000 Hz). In fact, more overtones were presented, whereas tonic tones became softer. At levels 3–4, the overtones started to dominate the acoustic sound pattern. The SPLs of lower frequencies were minimized, whereas the frequencies over 10 kHz became more compressed. Finally, the overtones dominated the acoustic sound pattern at level 5 and level 6 (the last 2 weeks of the intervention). Here, the low frequencies were no longer hearable for participants, and the compression of the high frequencies over 10 kHz reached its maximum. During the ASTM, children were allowed to pursue a silent activity, such as painting or reading picture books.

#### Pedagogical Activity Group and Controls

Children in the comparison group participated in a pedagogical activity program. Training lessons were organized in different group sizes, with a minimum of five and a maximum of ten children, depending on preschool capacities. The focus of the different activities was the preparation of primary school skills, such as visual differentiation and sequencing, set theory, the recognition of rhyming words and phoneme identification.

Children in the control group did not receive additional training at preschool. Nevertheless, after the intervention phase, those children had the choice to participate in the ASTM. No children received additional speech therapy before or during the intervention.

### Design

Children in the ASTM group were submitted to three 30-min weekly sessions over a period of 12 weeks. Children in the PA group were submitted to the program at comparable intervals. Moreover, the observation period of the children in the PA and CG groups between the tests at both time points of measure corresponded to that of the ASTM group. Sessions were organized in small groups (fewer than 10 children per session) in different rooms. Moreover, child care workers were instructed to administer a list indicating rate and duration of each intervention session to ensure that the exposure to either intervention was constant across groups. Data from children whose attendance was less than 80% were excluded from the analyses. Dependent variables were assessed at two different time points: before and after the intervention phase (Pre-Post-design).

The study design was approved by the institutional review board of the Carl von Ossietzky University of Oldenburg in Germany. Additional written informed consent for participation was obtained from preschool administration, parents and children included in the study.

### Procedure

To ensure high ecological validity, children were tested in groups in their preschool rooms during morning classes. Child care workers and preschool teachers conducted the interventions, whereas qualified examiners administered the test battery for data collection. To avoid teaching bias from care workers and preschool teachers and to minimize variability beyond the main experimental manipulation, each child care worker and preschool teacher conducted both types of intervention (AST and pedagogical training). The order of the instruments used at baseline was as follows: First, the language comprehension and working memory capacity of each child were measured via the *TROG-D* and the *HASE*. Second, further tests with regard to *Speech Perception* and *Phoneme Discrimination* skills were administered to a smaller number of children to ensure that the research protocol would not extend 45 min.

Twelve weeks later (Post-Test), each child in the study cohort repeated the HASE and the tests of their high frequency audibility on *Speech Perception* and *Phoneme Discrimination* (with and without background noise). To avoid novelty effects, the order of the *HASE*, the *Phoneme Discrimination Test* and the *Speech Perception Test* was the same for each child at both time points.

### Statistical Analyses

Repeated-measures analyses of variance (ANOVA) were conducted for the dependent measures of the auditory working memory (*Digit Span Test, Non-word Recall Test)*, the short-term processing of language (*Recall of Sentence Test)*, the *Phoneme Discrimination* (with and without noise) and the *Speech Perception* (4,000 Hz – 2,000 Hz) variables at two time points. The experimental design was a 3 × 2 mixed model, with Group (ASTM, PA and CG) as the between-subject factor and Time (pre-test *_(T__1__)_* vs. post-test *_(T__2__)_*) as the within-subject factor.

Preconditions for the ANOVAs with repeated measurement (normality, Box’s M test of equality of covariance matrices and Levene’s test of equality of variance) were tested for all dependent variables separately and were met in all cases. Moreover, *post hoc* tests were applied for multiple comparison of means via Bonferroni adjustments.

## Results

Table 1 reports the mean values and standard deviations for the independent and dependent variables for both the experimental and control groups.

**TABLE 1:** Means (and SD) of the auditory stimulation training group (ASTM), the pedagogical activity group (PA) and the control group (CG) for the measured variables at T1 and T2.

	**ASTM**		**PA**		**CG**	
			
**Measures**	**T1 M (SD)**	**T2 M (SD)**	**T1 M (SD)**	**T2 M (SD)**	**T1 M (SD)**	**T2 M (SD)**
Age	4.52 (0.64)	–	4.54 (0.59)	–	4.51 (0.56)	–
TROG-D	38.73 (3.24)	–	35.46 (4.45)	–	37.38 (3.97)	–
Digit span	2.63 (1.48)	3.63 (1.30)	2.63 (1.50)	2.79 (1.56)	2.78 (1.27)	3.03 (1.34)
Non-word recall	3.95 (1.57)	6.85 (1.66)	3.67 (1.61)	3.88 (1.85)	3.92 (1.48)	4.16 (1.50)
Recall of sentences	4.78 (1.90)	6.50 (1.43)	4.25 (2.42)	4.75 (2.05)	5.08 (1.77)	5.38 (2.03)
Phonemic discrimination (with noise)	7.44 (9.87)	27.00 (4.59)	11.92 (7.68)	13.92 (8.72)	11.65 (8.46)	10.91 (8.46)
Phonemic discrimination (without noise)	18.06 (9.93)	22.94 (5.51	17.25 (7.23)	21.75 (3.84)	16.54 (8.14)	18.04 (7.14)
Speech perception (4,000 Hz)	0.78 (1.81)	3.15 (2.60)	0.36 (0.50)	1.29 (1.86)	0.36 (0.76)	1.00 (1.82)
Speech perception (3,000 Hz)	2.59 (3.45)	7.44 (2.42)	1.64 (2.50)	5.29 (3.32)	2.75 (3.19)	3.96 (3.10)
Speech perception (2,000 Hz)	6.19 (4.11)	9.89 (0.32)	5.64 3(0.99)	9.21 (1.42)	5.54 (3.27)	7.00 (3.32)

### Auditory Screening Test: Working Memory Capacity and Short-Term Processing of Language

Analyses for the *Digit Span Test* revealed a significant group x time interaction (*F*_(2, 98)_ = 3.61, *p* = 0.03, η_*p*_^2^ = 0.07) and a main effect of time (*F*_(1, 98)_ = 10.18, *p* = 0.002, η_*p*_^2^ = 0.09). *Post hoc* analyses with pairwise comparisons at both time points showed that groups did not differ at *T*_1_ (*F*_(2, 98)_ = 0.15, *p* = 0.86) and *T*_2_ (*F*_(2, 98)_ = 3.24, *p* = 0.04). However, subsequent comparison of means for within-subject effects with a Bonferroni correction for the observed *p*-values (*p* = 0.05/3 = 0.016) showed that children in the ASTM group significantly improved their performances in the *Digit Span Test from T_1_ to T_2_* (*t*_(__39__)_ = 4.31, *p* < 0.001, *d* = 0.72), whereas no such increases were reported for the controls (PA group: *t*_(__23__)_ = 0.52, *p* = 0.61; CG: *t*_(__36__)_ = 1.12, *p* = 0.27).

With respect to the *Non-word Recall Test*, the results also showed a significant interaction (*F*_(2, 98)_ = 40.13, *p* < 0.001, η_*p*_^2^ = 0.45) and a main effect of group (*F*_(2, 98)_ = 13.11, *p* < 0.001, η_*p*_^2^ = 0.21) and time (*F*_(1, 98)_ = 55.85, *p* < 0.001, η_*p*_^2^ = 0.36). *Post hoc* test analyses showed no difference between groups at *T*_1_ (*F*_(2, 98)_ = 0.28, *p* = 0.76). However, children in the ASTM group (*M* = 6.85, *SD* = 1.66) outperformed children in the PA group (*M* = 3.88, *SD* = 1.85) and in the control group (*M* = 4.16, *SD* = 1.50) at T_2_ (*F*_(2, 98)_ = 35.18, *p* < 0.001, η_*p*_^2^ = 0.45). Within-subject effects revealed *that children in the ASTM* group significantly increased their *Non-word Recall Test score* from T_1_ to T_2_ (*t*_(39)_ = 10.47, *p* < 0.001, *d* = 1.80). No such increases were found for the controls (PA: *t*_(23)_ = 0.77, *p* = 0.45; CG: *t*_(36)_ = 1.25, *p* = 0.22).

The results for the *Recall of Sentences Test* showed a significant Group x Time interaction (*F*_(2, 98)_ = 12.55, *p* < 0.001, η_*p*_^2^ = 0.20). *Post hoc* tests observed no differences between groups at T1 (*F*_(2, 98)_ = 1.27, *p* = 0.29). However, the groups differed significantly at T_2_ (*F*_(2, 98)_ = 7.69, *p* = 0.001, η_*p*_^2^ = 0.42), with benefits for the children in the ASTM group (*M* = 6.50, *SD* = 1.43; PA group: *M* = 2.05, *SD* = 2.05; CG: *M* = 5.38, *SD* = 2.03) and children in the PA and CG groups.

Subsequent comparison of means for within-subject effects showed that children in the ASTM group (*t*_(39)_ = 7.92, *p* < 0.001, *d* = 0.98) and in the PA group (*t*_(23)_ = 2.51, *p* = 0.20, *d* = 0.21) significantly improved their performances for the *Recall of Sentences* from T_1_ to T_2_. However, the observed effect size was much higher for the children in the ASTM compared to the children in the PA group. No such increase was found for the children in the CG (*t*_(36)_ = 1.23, *p* = 0.23).

**FIGURE 2:**
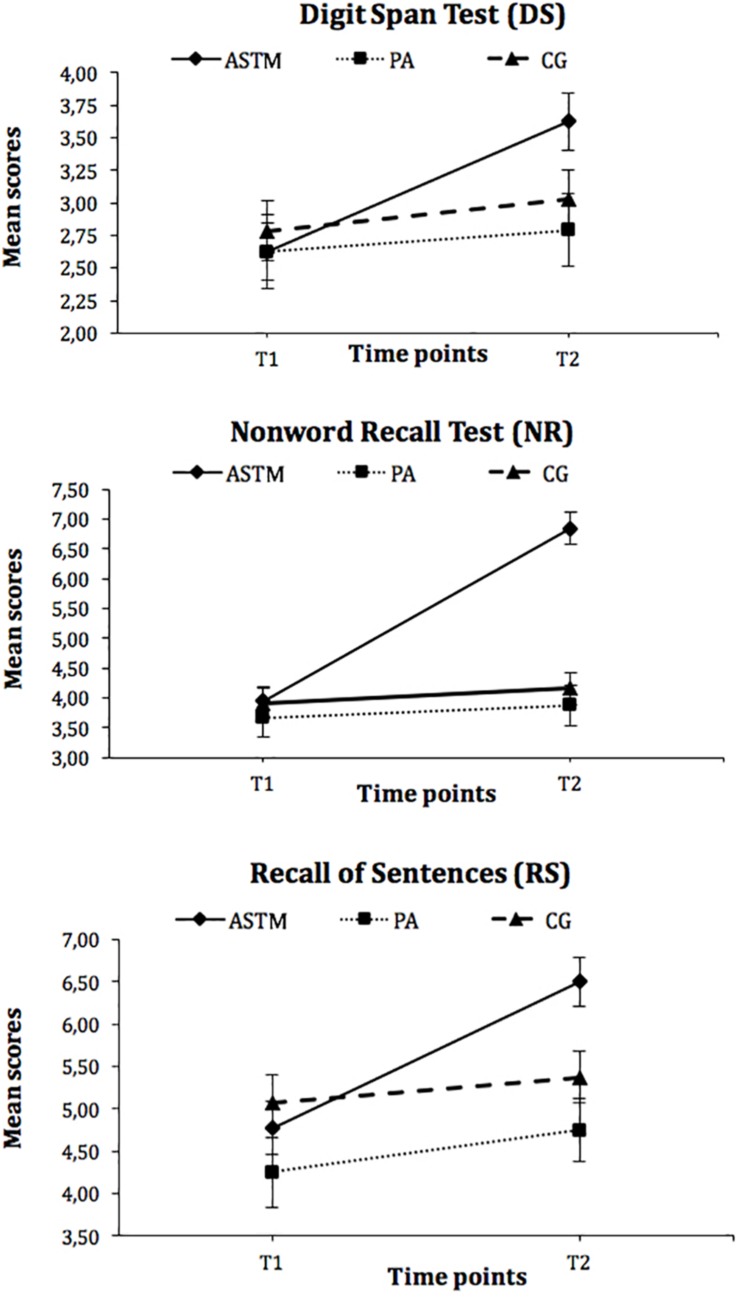
Mean scores of the auditory working memory tests (DS, NR, RS) for the auditory stimulation training with technically manipulated musical material group (ASTM), the pedagogical activity group (PA), and the control group (CG) at Tl and T2. Error flags indicate standard error of means (SEM).

The respective values for these working memory tests are displayed in Figure 2.

### Phoneme Discrimination Test With and Without Background Noise

**FIGURE 3:**
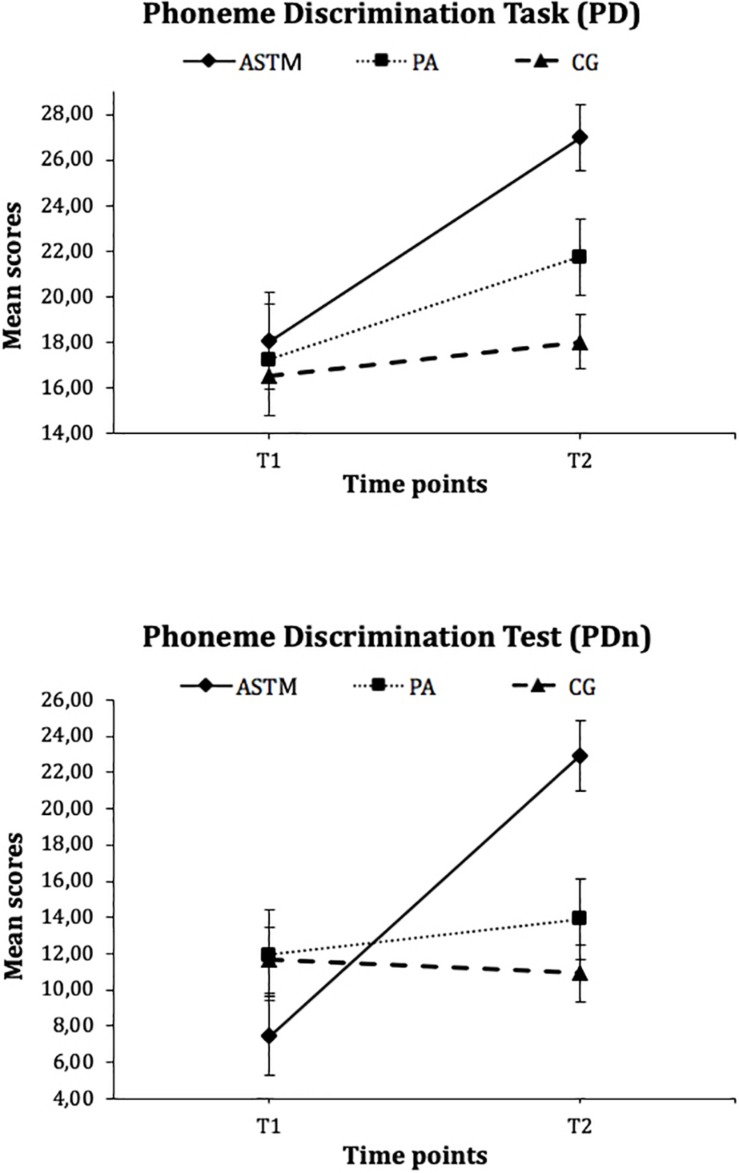
Mean scores of the Phoneme Discrimination Test (PD) and the Phoneme Discrimination Test with background noise (PDn) for the auditory stimulation training with technically manipulated musical material group (ASTM), the pedagogical activity group (PA), and the control group (CG) at Tl and T2. Error flags indicate standard error of means (SEM).

Figure 3 reveals a significant Group x Time interaction for the *Phoneme Discrimination Test* (PD) and for the *Phoneme Discrimination Test with background noise* (PDn) [PD: *F*_(2, 49)_ = 4.35, *p* = 0.02, η_*p*_^2^ = 0.15; *PD*_*n*_: *F*_(2, 48)_ = 21.06, *p* < 0.001, η_*p*_^2^ = 0.47]. *Post hoc* tests for the PD task showed no differences between groups at *T*_1_ (PD: *F*_(2, 49)_ = 0.15, *p* = 0.86). However, children in the ASTM group (*M* = 27.00, *SD* = 4.59) performed significantly better for the *PD* in comparison to the children in the PA group (*M* = 21.75, *SD* = 3.84) and in the CG (*M* = 18.04, *SD* = 7.14) at T_2_ (*F*_(2, 98)_ = 11.45, *p* < 0.001, η_*p*_^2^ = 0.69). Subsequent comparison of means showed that children in the ASTM group (*t*_(15)_ = 3.54, *p* = 0.003, *d* = 1.13) and in the PA group (*t*_(11)_ = 2.27, *p* = 0.04, *d* = 0.74) significantly improved their performances from T_1_ to T_2_, whereas children in the CG did not (*t*_(23)_ = 1.15, *p* = 0.26).

Consistent with the results of the *PD*, analyses for the *PD*_*n*_ revealed no difference between groups at T_1_ (*F*_(2, 48)_ = 1.33, *p* = 0.27), but at T_2_ (*F*_(2, 50)_ = 11.70, *p* < 0.001, η_*p*_^2^ = 0.67), there were benefits for the children in the ASTM group (ASTM group: *M* = 22.94, *SD* = 5.51; PA group: *M* = 13.92, *SD* = 8.72; CG: *M* = 10.91, *SD* = 8.46). Moreover, within-subject effects showed that children in the ASTM group significantly improved their skills from T_1_ to T_2_ (*t*_(15)_ = 8.50, *p* < 0.001, *d* = 1.69), whereas no such increase was found for the controls (PA group: *t*_(11)_ = 0.77, *p* = 0.46; CG: *t*_(22)_ = 0.46, *p* = 0.65).

### Speech Perception

**FIGURE 4:**
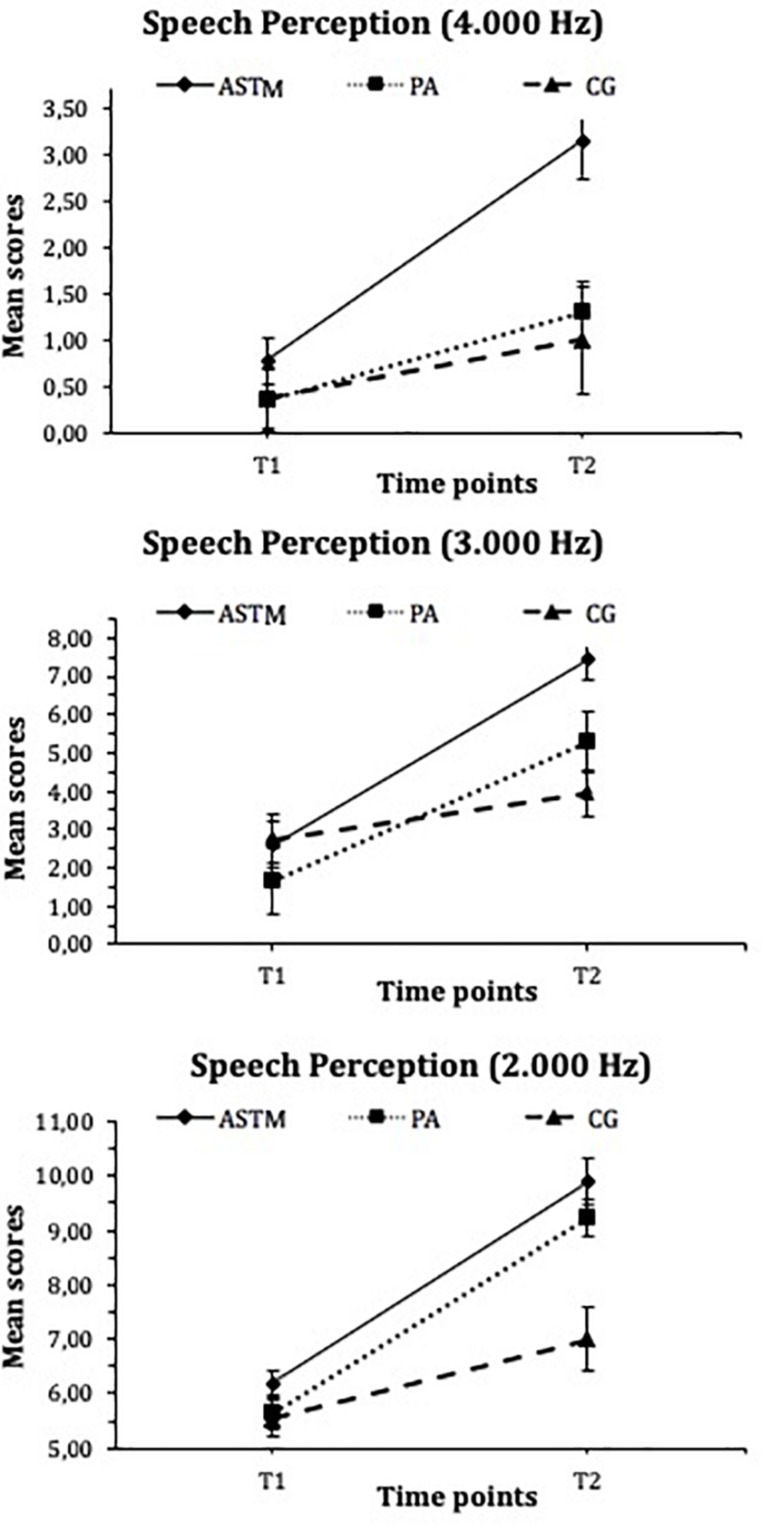
Mean scores for Speech Perception (4.000, 3.000, and 2.000 Hz] for the auditory stimulation training with technically manipulated musical material group (ASTM], the pedagogical activity group (PA], and the control group (CG) at Tl and T2. Error flags indicate standard error of means (SEM).

The final set of analyses addressed the *Speech Perception* skills (see Figure 4). The results for the *Speech Perception* at 4,000 Hz showed a significant interaction (*F*_(2, 62)_ = 6.97, *p* = 0.002, η_*p*_^2^ = 0.18) and a main effect of group (*F*_(2, 62)_ = 4.90, *p* = 0.011, η_*p*_^2^ = 0.14) and time (*F*_(1, 62)_ = 33.32, *p* < 0.001, η*_*p*_^2^* = 0.35). *Post hoc* tests showed no differences between groups for 4,000 Hz (*F*_(2, 64)_ = 0.87 *p* = 0.43) at *T*_1_. However, at T2 (*F*_(2, 64)_ = 6.97, *p* = 0.002, η_*p*_^2^ = 0.38), children in the ASTM group significantly outperformed the control group (ASTM group: *M* = 3.15, *SD* = 2.60; PA group: *M* = 1.29, *SD* = 1.86; CG: *M* = 1.00, *SD* = 0.18). Subsequent comparison of means showed that children in the ASTM group significantly increased their *Speech Perception* performances at 4,000 Hz (*t*_(26)_ = 6.15, *p* < 0.001, *d* = 1.01) from T_1_ to T_2_, whereas neither control did so (PA group: *t*_(13)_ = 2.18, *p* = 0.05; CG: *t*_(23)_ = 2.01, *p* = 0.06).

Moreover, the results for the *Speech Perception* at 3,000 Hz showed a significant interaction (*F*_(2, 62)_ = 8.30, *p* < 0.001, η_*p*_^2^ = 0.21) and a main effect of group (*F*_(2, 62)_ = 3.15, *p* = 0.04, η_*p*_^2^ = 0.09) and time (*F*_(1, 62)_ = 60.70, *p* < 0.001, η_*p*_^2^ = 0.50). *Post hoc* analyses revealed no difference between groups at *T*_1_ (*F*_(2, 64)_ = 0.59 *p* = 0.56). However, children in the ASTM group significantly outperformed their controls at *T*_2_ (*F*_(2, 64)_ = 9.45 *p* < 0.001, η_*p*_^2^ = 0.05; ASTM group: *M* = 7.44, *SD* = 2.42; PA group: *M* = 5.29, *SD* = 3.32; CG: *M* = 3.96, *SD* = 3.01). Within-subject effects revealed that children in the ASTM group (*t*_(26)_ = 7.61, *p* < 0.001, *d* = 1.60) and in the PA group (*t*_(13)_ = 4.79, *p* < 0.001, *d* = 1.21) had significantly improved their *Speech Perception* at 3,000 Hz. However, reported effect size was higher for the children in the ASTM group. No increase from T_1_ to T_2_ was found for the children in the CG (*t*_(23)_ = 1.80, *p* = 0.09).

Finally, *Speech Perception* at 2,000 Hz showed a significant main effect of group (*F*_(2, 62)_ = 4.90, *p* = 0.011, η_*p*_^2^ = 0.14) and time (*F*_(1, 62)_ = 33.32, *p* < 0.001, η_*p*_^2^ = 0.35) but no significant interaction (*F*_(2, 62)_ = 2.66, *p* = 0.08). *Post hoc* analyses revealed that groups did not differ at *T*_1_ (*F*_(2, 64)_ = 0.20 *p* = 0.82). However, children in the ASTM (*M* = 9.89, *SD* = 0.32) and PA groups (M = 9.21, SD = 0.142) performed higher than did children in the CG (*M* = 7.00, *SD* = 3.32) at T2 (*F*_(2, 64)_ = 12.18 *p* < 0.001, η_*p*_^2^ = 0.41). Moreover, subsequent comparison of means for within-subject effects showed that children in the ASTM group (*t*_(26)_ = 4.87, *p* < 0.001, *d* = 0.90) and in the PA group (*t*_(13)_ = 3.70, *p* = 0.003, *d* = 1.05) improved their *Speech Perception* performances at 2,000 Hz from T_1_ to T_2_. Children in the CG did not show a significant increase (*t*_(23)_ = 2.05, *p* = 0.05).

Finally, correlation analyses showed a positive relationship among the auditory working memory, the *Phoneme Discrimination* and the *Speech Perception* tasks (see Table 2).

**TABLE 2:** Correlations (r) between dependent variables at T2.

	**RS**	**NR**	**SP 4,000 Hz**	**SP 3,000 Hz**	**SP 2,000 Hz**	**PD**	**PD_*n*_**
Digit span (DS)	0.50^∗∗^	0.40^∗∗^	0.17	0.23	0.11	0.41^∗∗^	0.22
Recall of sentences (RS)		0.51^∗∗^	0.33	0.38^∗∗^	0.24	0.40	0.40
Non-word recall (NR)			0.39^∗∗^	0.53^∗∗^	0.48^∗∗^	0.60^∗∗^	0.43^∗∗^
Speech perception (SP) at 4,000 Hz				0.64^∗∗^	0.41^∗∗^	0.41^∗∗^	0.51^∗∗^
Speech perception (SP) at 3,000 Hz					0.72^∗∗^	0.46^∗∗^	0.62^∗∗^
Speech Perception (SP) at 2,000 Hz						0.45^∗∗^	0.37
Phoneme discrimination (PD)							0.55^∗∗^

*PD_*n*_ = Phoneme Discrimination with background noise. Due to the risk of a type I error for calculating numerous correlations, *p*-value for all correlations was adjusted to *p* < 0.002 (see Cupples et al., 1984). ^∗∗^The correlation is significant at the level of 0.002.*

However, calculating numerous correlations increases the risk of a type I error. In order to avoid this and to keep the overall α-level at 5%, we used an approach by Šidák (1967), in which the adjusted α′-level could be calculated exactly: α′=1−(1−*overall* α) ^1/*k*^. The letter ‘*k*′ represents the number of correlation coefficients that were calculated from the data. Thus, the adjusted α′-level for the multiple correlations reported in Table 2 was = 0.002. In particular, Pearson’s product correlation coefficient revealed a moderate correlation between the *Non-word Recall Test* and the speech perception outcomes for all groups at *T*_2_ (4,000 Hz: *r* = 0.39; 3,000 Hz: *r* = 0.53; 2,000 Hz: *r* = 0.48, all *p*-values <0.002). ANCOVAs with repeated measurement for the *Speech Perception* outcomes and the means of the *Non-word Recall Test* as covariate were assessed. The interaction effect remained significant at 3,000 Hz (*F*_(2, 61)_ = 3.62, *p* = 0.03, η_*p*_^2^ = 0.11), whereas the interaction effect at 4,000 Hz and 2,000 Hz was no longer significant (4,000 Hz: *F*_(2, 61)_ = 1.77, *p* = 0.18; 2,000 Hz: *F*_(2, 61)_ = 2.45, *p* = 0.09). Complete correlation tables for each group are attached in the Supplementary Appendix 1.

## Discussion

In the current study, we investigated whether AST with musical material influences auditory working memory performance, language processing, phoneme discrimination and high frequency hearing abilities in preschool children with SLI. We hypothesized that children in the ASTM group would outperform their controls in each of these tasks. A randomized control trial with pre-post-measurements over a period of 12 weeks of intervention was designed to address these assumptions. We will discuss the findings for each of the three dependent measures in turn.

### ASTM, Working Memory Capacity and Short-Term Processing of Language (H1)

First, we found that children in the ASTM group scored significantly higher on auditory working memory and short-term processing of language measures after the intervention than the controls. Specifically, children in the ASTM group revealed significant increases in their scores on the *Digit Span*, the *Non-word Recall*, and the *Recall of Sentences* tests, whereas the comparison and control group showed no such improvement (except the PA group for the *Recall of Sentences*). The percentage increase for the ASTM group was between 26% for the *Recall of Sentences Test*, 27% for the *Digit Span* test and 42% for the *Non-word Recall Test*. These findings suggest that ASTM enhances the phonological working memory capacity. Moreover, our results are consistent with previous studies showing that typically developed children benefit from music training to improve their auditory working memory abilities (Lee et al., 2007; Roden et al., 2012). They extend previous findings by suggesting that ASTM might also develop the phonological short term memory and phonological coding strategies of written words of children with SLI. Given the high relevance of phonological working memory for the development of both language and literacy, the observed results are quite remarkable, especially when we consider that deficits in phonological working memory usually persist in children with delayed language development even after successful speech therapy (Henry, 2012). But also when we assume that previous research suggests that children with dyslexia - in contrast to typically developed children – often rely on visual instead of phonological coding strategies for the mediation of words in working memory (Kast et al., 2011).

### ASTM and Phoneme Discrimination (H2)

Second, children in the ASTM group also benefited in terms of phoneme discrimination abilities. Whereas there were no significant differences between groups at baseline, children in the ASTM group outperformed their peers in the control groups at the end of the intervention. In addition, children in the ASTM group increased their performance significantly over time, whereas controls did not. One reason for these findings might be the close relationship between phoneme discrimination abilities and formant detection on the one hand and the similarity of formants (in speech) and resonance (in music) on the other hand. This interpretation is consistent with previous research suggesting a link between sound discrimination deficits in children with SLI and the inability to perceive formant transitions (Overy et al., 2003; Filippini et al., 2012; Heim et al., 2013).

### ASTM and Speech Perception (H3)

Finally, the data from the present study suggest an advantageous effect of AST with musical material on the speech perception performances at high frequencies (4,000 Hz to 2,000 Hz), where children in the ASTM group outperformed controls. Moreover, children in the ASTM group showed a significantly higher increase from T1 to T2 for their speech perception performances at 4,000 Hz, 3,000 Hz and 2,000 Hz, whereas children in the PA group improved their scores only at 3,000 and 2,000 Hz. In contrast, children in the control group did not increase their performances over time. Several aspects of the ASTM might have caused those results. First, the intervention focused on the training of the perception of speech presented at high frequencies above 2,000 Hz, which are essential for speech perception. Therefore, original music pieces were filtered to remove frequencies below a) 4,000 Hz, b) 3,000 Hz, and c) 2,000 Hz. Hence, the ASTM might have improved the perception of higher frequency for the children in the intervention group. Second, the sound delivery was lateralized such that frequencies presented to the left ear at higher levels were attenuated to the right ear and vice versa. This processing might lead to an improved coordination of the cerebral hemisphere, which is crucial for speech processing. Furthermore, the lateralization training might have increased the precision of the processing time for sound and phoneme discrimination (Ryding et al., 1996; Ross et al., 1997).

We further explored whether the improved performances of *Speech Perception* lead to higher auditory working memory performance. Therefore, we investigated the relationship between phonological short term memory and the *Speech Perception outcomes* for all groups. Pearson correlations revealed highly significant correlations of moderate sizes between the Speech Perception at 4,000, 3,000, and 2,000 Hz and the *Non-word Recall Test* at T2. Finally, ANCOVAs with repeated measurement for the *Speech Perception* outcomes (at 4,000 Hz and 3,000 Hz) and the means of the *Non-word Recall* were assessed. Interestingly, statistical analyses revealed different results. Whereas the interaction term remained significant in the analyses for the *Speech Perception Test* at 3,000 Hz, the interaction effect at 4,000 Hz and 2,000 Hz failed to reach significance. Hence, further research will be necessary to clarify which training aspects of *Speech Perception* might affect the phonological short term memory. Moreover, the highly significant relationship between phoneme discrimination abilities and the perception of speech (all *r*’s > 0.41, all *p*-values <0.001) might be explained by the fact that the detection of single formants and phonemes takes place between 4,000 and 3,000 Hz.

Considering the significant interaction effects with high effect sizes for all dependent variables (except for *Speech Perception* at 2,000 Hz) and the fact that the three groups did not differ at *pre*-tests for any of the eight dependent measures, our results suggest some validation of a transfer effect of ASTM with more-specific cognitive domains, including components of auditory working memory, short-term processing of language, phoneme discrimination and speech perception abilities. Moreover, this pattern of findings suggests that the randomly assigned groups of children were well distributed at the beginning of the intervention phase and that the superior performance by the ASTM group on the dependent variables at *post*-test measuring can be attributed to the effects of the ASTM. To our knowledge, this study is the first to show the benefits of an ASTM in preschool children based on a randomized control group design, producing even more persuasive evidence than prior quasi-experimental approaches.

Moreover, it supports previous findings from electrophysiological and behavioral research suggesting that the training of non-linguistic auditory and audiovisual stimuli might cause changes in the neural substrate of sound discrimination in children with dyslexia (Kujala et al., 2001; Kast et al., 2007, 2011).

## Limitations and Conclusion

The current study must be interpreted with some caveats in mind. First, one might argue that the reported benefits for children in the ASTM group over 12 weeks of intervention might be related to a regression to the mean effect or in favor of power for the ASTM group. However, because the current study is based on a randomized control group design and differences between groups in dependent measures at baseline were not significant, this issue appears negligible. Second, the ASTM includes several aspects that might have affected the observed variables, such as the training of the perception of high frequencies or the lateralization training of hemispheric coordination. To clarify which aspects of the ASTM might have caused the observed effects, further investigations are necessary that focus, for example, on neuropsychological mechanisms such as mismatch negativity or other neuroimaging procedures. Moreover, the improvement of language-related outcomes after ASTM might been related to indirectly trained skills, such as cognitive skills, rather than auditory temporal processing (see Gaab et al., 2007).

Third, compared to the individual speech therapies for children with SLI, in which the auditory and phonological deficits persist despite successful treatment (Henry, 2012), the ASTM appeared to be very efficient. However, whether those observed effects are sustainable requires further investigations with additional follow-up tests that include retention intervals after the ASTM intervention. Fourth, some might argue that children in the ASTM group were allowed to paint and read during the intervention, which might substantially alter the amount of attention the children devoted to listening to music. Nevertheless, children in the ASTM group showed significant correlations for all the dependent *pre-post*-test measures, which provides additional evidence that the advantage for the ASTM group children relies on the specific experience of listening to music. Fifth, the group size between the ASTM and PA groups varies from 5–6 participants in the ASTM group to 5–10 participants in the PA group. However, group size for both intervention groups was relatively small, and none of the participants received individual training lessons. Thus, it seems unlikely that the different group size between the ASTM and PA groups would account for our results in total. According to the different size of all three groups (ASTM: *n* = 40; PA: *n* = 24; CG: *n* = 37), however, a balanced design would lead to more powerful analysis by decreasing the type II error and strengthen its resistance to violations (Milhken and Johnson, 1984). Finally, further studies might control for any differences in the musical aptitude of the participants to clarify additional influences on the observed effects. Moreover, future studies should take into account that an additional control group in which the unfiltered musical material is be played could further clarify whether the observed effects are based on the ASTM or on music listening. In the current study, we refrained from installing such a comparison group for two main reasons, one theoretic, one pragmatic. First, the literature does not provide sufficient evidence to hypothesize that music listening *per se* could have positive effects in the rehabilitation of children with SLI. Second, at the time of the intervention, access to children who were eligible to participate in this study was limited. Therefore, we decided to run an alternative (pedagogical) intervention group rather than music listening.

In conclusion, the present study provides preliminary evidence that AST with musical material might positively affect auditory working memory capacities, phoneme discrimination performance and speech processing in children with SLI. Our study further strengthens previous results by confirming them in a randomized *pre-post* study design over 12 weeks of intervention. Although the mechanism that drives these effects remains unclear, the findings are consistent with previous approaches focusing on the relevance of auditory processing disorders in children with SLI. However, further studies should include a group of children who experienced music that was not acoustically modified to assess whether the reported effects are due to the acoustic modifications. Nevertheless, the last decade of examinations on the effects of listening to music on cognitive abilities reports effects that are either null or small, suggesting that music listening might have a much smaller potential compared to AST with technically manipulated musical material (see Kampfe et al., 2010).

In summary, our findings indicate an important first step toward showing that auditory cognitive processing and working memory affect language functioning and promote language development in children with SLI. Therefore, we suggest that ASTM could be used as a supplement to speech therapy in language development disorders or in educational institutions for language promotion.

## Ethics Statement

The study was approved by the ethics committee of the Carl von Ossietzky University of Oldenburg, Germany.

## Author Contributions

IR analyzed the data and assisted the research design process. IR and GK wrote the manuscript. KF, FL, and DG planned the research design of the study and were responsible for the collection of the data.

## Conflict of Interest Statement

The authors declare that the research was conducted in the absence of any commercial or financial relationships that could be construed as a potential conflict of interest.

## Footnotes

1 http://www.audiva.de/en-products-HPT-versions.html

## References

[B1] Agnew J. A. Dorn C. Eden G. F. (2004). Effect of intensive training on auditory processing and reading skills. *Brain Lang.* 88 21–25. 10.1016/S0093-934X(03)00157-3 14698727

[B2] Alexander D. W. Frost B. P. (1982). decelerated synthesized speech as a means of shaping speed of auditory processing of children with delayed language. *Percept. Mot. Skills* 55 783–792. 10.2466/pms.1982.55.3.783 6219342

[B3] Baddeley A. D. (2006). “Working memory: an overview,” in *Working Memory and Education*, ed. Pickering S. (New York, NY: Academic Press).

[B4] Bishop D. V. (1989). *Test for the Reception of Grammar (TROG)*, 2nd Edn United Kingdom: Medical Research Council.

[B5] Bishop D. V. M. Adams C. V. Rosen S. (2006). Resistance of grammatical impairment to computerized comprehension training in children with specific and non-specific language impairments. *Int. J. Lang. Commun. Dis.* 41 19–40. 10.1080/13682820500144000 16272001

[B6] Brandt A. Gebrian M. Slevc L. R. (2012). Music and early language acquisition. *Front. Psychol.* 3:327. 10.3389/fpsyg.2012.00327 22973254 PMC3439120

[B7] Lindamood Bell Learning Processes, (1999). *Lindamood Phoneme Sequencing Program [Computer Software].* San Luis Obispo, CA: Lindamood Bell Learning Processes.

[B8] Clément S. Planchou C. Béland R. Motte J. Samson S. (2015). Singing abilities in children with Specific Language Impairment (SLI). *Front. Psychol.* 6:420. 10.3389/fpsyg.2015.00420 25918508 PMC4394662

[B9] Cohen W. Hodson A. O’Hare A. Boyle J. Durrani T. McCartney E. (2005). Effects of computer-based intervention through acoustically modified speech (Fast For Word) in severe mixed receptive–expressive language impairment. *J. Speech Lang. Hear. Res.* 48 715–729. 10.1044/1092-4388(2005/049) 16197283

[B10] Cognitive Concepts, (1996). *Earobics [Computer Software].* Evanston IL: Cognitive Concepts.

[B11] Cognitive Concepts, (1997). *Earobics: Auditory Development and Phonics Program [Computer Software].* Cambridge MA: Cognitive Concepts.

[B12] Corriveau K. Pasquini E. Goswami U. (2007). Basic auditory processing skills and specific language impairment: a new look at an old hypothesis. *J. Speech Lang. Hear. Res.* 50 647–666. 10.1044/1092-4388(2007/046) 17538107

[B13] Corriveau K. H. Goswami U. (2009). Rhythmic motor entrainment in children with speech and language impairments: tapping to the beat. *Cortex* 45 119–130. 10.1016/j.cortex.2007.09.008 19046744

[B14] Cumming R. Wilson A. Leong V. Colling L. J. Goswami U. (2015). Awareness of rhythm patterns in speech and music in children with specific language impairments. *Front. Hum. Neurosci.* 9:672. 10.3339/fnhum.2015.00672 26733848 PMC4686839

[B15] Cupples L. A. Heeren T. Schatzkin A. Colton T. (1984). Multiple testing of hypotheses in comparing two groups. *Ann Int. Med.* 100 122–129. 6691637 10.7326/0003-4819-100-1-122

[B16] Dege F. Schwarzer G. (2011). The effect of a music program on phonological awareness in preschoolers. *Front. Psychol.* 2:124. 10.3389/fpsyg.2011.00124 21734895 PMC3121007

[B17] Ecalle J. Magnan A. Bouchafa H. Gombert J. E. (2009). Computer-based training with ortho-phonological units in dyslexic children: new investigations. *Dyslexia* 15 218–238. 10.1002/dys.373 18646049

[B18] Erdfelder E. Faul F. Buchner A. (1996). GPOWER: a general power analysis program. *Behav. Res. Methods Instrum. Comput.* 28 1–11. 10.3758/BF03203630

[B19] Fey M. E. Richard G. J. Geffner D. Kamhi A. G. Medwetsky L. Paul D. (2011). Auditory processing disorder and auditory/language interventions: an evidence-based systematic review. *Lang. Speech Hear. Serv. Sch.* 42 246–264. 10.1044/0161-1461(2010/10-0013) 20844275

[B20] Filippini R. Befi-Lopes D. M. Schochat E. (2012). Efficacy of auditory training using the auditory brainstem response to complex sounds: auditory processing disorder and specific language impairment. *Folia Phoniatr. Logop.* 64 217–226. 10.1159/000342139 23006808

[B21] Fisher M. Holland C. Merzenich M. M. Vinogradov S. (2009). Using neuroplasticity-based auditory training to improve verbal memory in schizophrenia. *Am. J. Psychiatry* 166 805–811. 10.1176/appi.ajp.2009.08050757 19448187 PMC2720319

[B22] Fitch R. H. Miller S. Tallal P. (1997). Neurobiology of speech perception. *Ann. Rev. Neurosci.* 20 331–353. 10.1146/annurev.neuro.20.1.331 9056717

[B23] Flaugnacco E. Lopez L. Terribili C. Zoia S. Buda S. Tilli S. (2014). Rhythm perception and production predict reading abilities in developmental dyslexia. *Front. Hum. Neurosci.* 8:392. 10.3389/fnhum.2014.00392 24926248 PMC4045153

[B24] Fonseca-Mora M. C. Jara-Jiménez P. Gómez-Domínguez M. (2015). Musical plus phonological input for young foreign language readers. *Front. Psychol.* 6:286. 10.3389/fpsyg.2015.00286 25852604 PMC4362048

[B25] Francois C. Chobert J. Besson M. Schon D. (2013). Music training for the development of speech segmentation. *Cereb. Cortex* 23 2038–2043. 10.1093/cercor/bhs180 22784606

[B26] Franssen V. Vandierendonck A. Van Hiel A. (2006). Duration estimation and the phonological loop: articulatory suppression and irrelevant sounds. *Psychol. Res.* 70 304–316. 10.1007/s00426-005-0217-x 16001277

[B27] Friel-Patti S. DesBarres K. Thibodeau L. (2001). Case studies of children using fast forword. *Am. J. Speech Lang. Pathol.* 10 203–215. 10.1044/1058-0360(2001/019)

[B28] Gaab N. Gabrieli J. D. E. Deutsch G. K. Tallal P. Temple E. (2007). Neural correlates of rapid auditory processing are disrupted in children with developmental dyslexia and ameliorated with training: an fMRI study. *Restor. Neurol. Neurosci.* 25 295–310. 17943007

[B29] Gathercole S. E. (1996). *Children’s Test of Nonword Repetition.* San Antonio, TX: The Psychological Corporation.

[B30] Gil D. Iorio M. C. (2010). Formal auditory training in adult hearing aid users. *Clinics* 65 165–174. 10.1590/S1807-59322010000200008 20186300 PMC2827703

[B31] Gillam R. B. Crofford J. A. Gale M. A. Hoffman L. M. (2001). Language change following computer-assisted language instruction with fast forword or laureate learning systems software. *Am. J. Speech Lang. Pathol.* 10 231–247. 10.1044/1058-0360(2001/021)

[B32] Goh C. Taib Y. (2006). Metacognitive instruction in listening for young learners. *ELT J.* 60 222–232. 10.1093/elt/ccl002

[B33] Gordon R. L. Fehd H. M. McCandliss B. D. (2015). Does music training enhance literacy skills? A meta-analysis. *Front. Psychol.* 6:1777. 10.3389/fpsyg.2015.01777 26648880 PMC4664655

[B34] Goswami U. (2010). “Language, music, and children’s brains: a rhythmic timing perspective on language and music as cognitive systems,” in *Language and Music as Cognitive Systems*, eds Rebuschat P. Rohrmeier M. John A. Hawkins I. C., (Oxford: Oxford University Press), 292–301. 10.1093/acprof:oso/9780199553426.003.0030

[B35] Habib M. (2000). The neurological basis of developmental dyslexia: an overview and working hypothesis. *Brain* 123 2373–2399. 10.1093/brain/123.12.2373 11099442

[B36] Hannon E. E. Johnson S. P. (2005). Infants use meter to categorize rhythms and melodies: implications for musical structure learning. *Cogn. Psychol.* 50 354–377. 10.1016/j.cogpsych.2004.09.003 15893524

[B37] Hasselhorn V. M. Schumann-Hengsteler R. Gronauer J. Grube D. Mähler C. Schmid I. (2012). *Arbeitsgedächtnistestbatterie für Kinder Von 5 Bis 12 Jahren: AGTB 5-12.* Boston, MA: Hogrefe.

[B38] Hayes E. A. Warrier C. M. Nicol T. G. Zecker S. G. Kraus N. (2003). Neural plasticity following auditory training in children with learning problems. *Clin. Neurophysiol.* 114 673–684. 10.1016/s1388-2457(02)00414-5 12686276

[B39] Heim S. Keil A. Choudhury N. Friedman J. T. Benasich A. A. (2013). Early gamma oscillations during rapid auditory processing in children with a language-learning impairment: changes in neural mass activity after training. *Neuropsychologica* 51 990–1001. 10.1016/j.neuropsychologia.2013.01.011 23352997 PMC3633611

[B40] Henry L. (2012). *The Development of Working Memory in Children. Discoveries & Explanations in Child Development.* Thousand Oaks, CA: Sage.

[B41] Huss M. Verney J. P. Fosker T. Mead N. Goswami U. (2011). Music, rhythm, rise time perception and developmental dyslexia: perception of musical meter predicts reading and phonology. *Cortex* 47 674–689. 10.1016/j.cortex.2010.07.010 20843509

[B42] Kampfe J. Sedlmeier P. Renkewitz F. (2010). The impact of background music on adult listeners: a meta-analysis. *Psychol. Music* 39 424–448. 10.1177/0305735610376261

[B43] Kast M. Baschera G. M. Gross M. Jäncke L. Meyer M. (2011). Computer-based learning of spelling skills in children with and without dyslexia. *Ann. Dyslexia* 61 177–200. 10.1007/s11881-011-0052-2 21562919

[B44] Kast M. Meyer M. Vögeli C. Gross M. Jäncke L. (2007). Computer-based multisensory learning in children with developmental dyslexia. *Restor. Neurol. Neurosci.* 25 355–369. 17943011

[B45] Kujala T. Karma K. Ceponiene R. Belitz S. Turkkila P. Tervaniemi M. (2001). Plastic neural changes and reading improvement caused by audiovisual training in reading-impaired children. *Natl. Acad. Sci.* 98 10509–10514. 10.1073/pnas.181589198 11517333 PMC56991

[B46] Lee Y. Lu M. Ko H. (2007). Effects of skill training on working memory capacity. *Learn. Instr.* 17 336–344. 10.1016/j.learninstruc.2007.02.010

[B47] Loeb D. F. Stoke C. Fey M. E. (2001). Language changes associated with fast forword-language. *Am. J. Speech Lang. Pathol.* 10 216–230. 10.1044/1058-0360(2001/020) 29364620

[B48] Megale R. L. Iorio M. C. Schochat E. (2010). Auditory training: assessment of the benefit of hearing aid in elderly individuals. *Pró Fono* 22 101–106. 20640372 10.1590/s0104-56872010000200006

[B49] Milhken G. A. Johnson D. E. (1984). *Analysis of Messy Data. Volume 1: Designed Experiments.* New York, NY: Van Nostrand Reinhold.

[B50] Miranda E. C. Gil D. Iorio M. C. (2008). Formal auditory training in elderly hearing aid users. *Braz. J. Otorhinolaryngol.* 74 919–925. 10.1016/S1808-8694(15)30154-3 19582350 PMC9445905

[B51] Murphy C. F. B. Schochat E. (2013). Effects of different types of auditory temporal training on language skills: a systematic review. *Clinics* 68 1364–1370. 10.6061/clinics/2013(10)12 24212845 PMC3798554

[B52] Overy K. (2000). Dyslexia, temporal processing and music: the potential of music as an early learning aid for dyslexic children. *Psychol. Music* 28 218–229. 10.1177/0305735600282010

[B53] Overy K. Nicolson R. I. Fawcett A. J. Clarke E. F. (2003). Dyslexia and music: measuring musical timing skills. *Dyslexia* 9 18–36. 10.1002/dys.233 12625374

[B54] Planchou C. Clément S. Béland R. Cason N. Motte J. Samson S. (2015). Word detection in sung and spoken sentences in children with typical language development or with specific language impairment. *Adv. Cogn. Psychol.* 11 118–135. 10.5709/acp-0177-8 26767070 PMC4710888

[B55] Pokorni J. L. Worthington C. K. Jamison P. J. (2004). Phonological awareness intervention: comparison of fast forword, earobics, and lips. *J. Educ. Res.* 97 147–158. 10.2307/27548023 18230858

[B56] Przybylski L. Bedoin N. Krifi-Papoz S. Herbillon V. Roch D. Léculier L. (2013). Rhythmic auditory stimulation influences syntactic processing in children with developmental language disorders. *Neuropsychology* 27 121–131. 10.1037/a0031277 23356600

[B57] Reilly S. Bishop D. V. Tomblin B. (2014). Terminological debate over language impairment in children: forward movement and sticking points. *Int. J. Lang. Commun. Dis.* 49 452–462. 10.1111/1460-6984.12111 25142092 PMC4312775

[B58] Roden I. Grube D. Bongard S. Kreutz G. (2014). Does music training enhance working memory performance? Findings from a quasi-experimental longitudinal study. *Psychol. Music* 42 284–298. 10.1177/0305735612471239

[B59] Roden I. Kreutz G. Bongard S. (2012). Effects of a school-based instrumental music program on verbal and visual memory in primary school children: a longitudinal study. *Front. Psychol.* 3:572. 10.3389/fpsyg.2012.00572 23267341 PMC3528082

[B60] Ross E. D. Thompson R. D. Yenkosky J. (1997). Lateralization of affective prosody in brain and the callosal integration of hemispheric language functions. *Brain Lang.* 56 27–54. 10.1006/BRLN.1997.1731 8994697

[B61] Russo N. M. Nicol T. G. Zecker S. G. Hayes E. A. Kraus N. (2005). Auditory training improves neural timing in the human brainstem. *Behav. Brain Res.* 156 95–103. 10.1016/j.bbr.2004.05.012 15474654

[B62] Ryding E. Brådvik B. Ingvar D. H. (1996). Silent speech activates prefrontal cortical regions asymmetrically, as well as speech-related areas in the dominant hemisphere. *Brain Lang.* 52 435–451. 10.1006/brln.1996.0023 8653389

[B63] Sallat S. Jentschke S. (2015). Music perception influences language acquisition: melodic and rhythmic-melodic perception in children with specific language impairment. *Behav. Neurol.* 2015:606470. 10.1155/2015/606470 26508812 PMC4610061

[B64] Schochat E. Musiek F. E. Alonso R. Ogata J. (2010). Effect of auditory training on the middle latency response in children with (central) auditory processing disorder. *Braz. J. Med. Biol. Res.* 43 777–785. 10.1590/s0100-879x2010007500069 20658093

[B65] Schöler H. Brunner M. (2009). *Heidelberger Auditives Screening in Der Einschulungsuntersuchung.* Franeker: Westra.

[B66] Scientific Learning Corporation, (1998). *Fast For Word [Computer Software].* Berkeley, CA: Scientific Learning Corporation.

[B67] Segers E. Verhoeven L. (2004). Computer-supported phonological awareness intervention for kindergarten children with specific language impairment. *Lang. Speech Hear. Serv. Sch.* 35 229–239. 10.1044/0161-1461(2004/022) 15248793

[B68] Šidák Z. (1967). Rectangular confidence regions for the means of multivariate normal distributions. *J. Am. Stat. Assoc.* 62 626–633. 10.1080/01621459.1967.10482935

[B69] Steinbrink C. Knigge J. Mannhaupt G. Sallat S. Werkle A. (2019). Are Temporal and tonal musical skills related to phonological awareness and literacy skills?–Evidence from two cross-sectional studies with children from different age groups. *Front. Psychol.* 10:805. 10.3389/fpsyg.2019.00805 31040806 PMC6477020

[B70] Steinbrink C. Zimmer K. Lachmann T. Dirichs M. Kammer T. (2014). Development of rapid temporal processing and its impact on literacy skills in primary school children. *Child Dev.* 85 1711–1726. 10.1111/cdev.12208 24359600

[B71] Stevens C. Fanning J. Coch D. Sanders L. Neville H. (2008). Neural mechanisms of selective auditory attention are enhanced by computerized training: electrophysiological evidence from language-impaired and typically developing children. *Brain Res.* 1205 55–69. 10.1016/j.brainres.2007.10.108 18353284 PMC2426951

[B72] Strehlow U. Haffner J. Bischof J. Gratzka V. Parzer P. Resch F. (2006). Does successful training of temporal processing of sound and phoneme stimuli improve reading and spelling? *Eur. Child Adolesc. Psychiatry* 15 19–29. 10.1007/s00787-006-0500-4 16514506

[B73] Tallal P. (1980). Auditory temporal perception, phonics, and reading disabilities in children. *Brain Lang.* 9 182–198. 10.1016/0093-934x(80)90139-x 7363063

[B74] Tallal P. Miller S. L. Bedi G. Byma G. Wang X. Nagarajan S. S. (1996). Language comprehension in language-learning impaired children improved with acoustically modified speech. *Science* 271 81–84. 10.1126/science.271.5245.81 8539604

[B75] Tallal P. Piercy M. (1973). Defects of non-verbal auditory perception in children with developmental aphasia. *Nature* 241 468–469. 10.1038/241468a0 4705758

[B76] Thomson J. M. Goswami U. (2008). Rhythmic processing in children with developmental dyslexia: auditory and motor rhythms link to reading and spelling. *J. Physiol.Paris* 102 120–129. 10.1016/j.jphysparis.2008.03.007 18448317

[B77] Tierney A. Kraus N. (2013). Music training for the development of reading skills. *Prog. Brain Res.* 207 209–241. 10.1016/B978-0-444-63327-9.00008-4 24309256

[B78] Vilela N. Wertzner H. F. Sanches S. G. G. Neves-Lobo I. F. Carvallo R. M. M. (2012). Processamento temporal de crianças com transtorno fonológico submetidas ao treino auditivo: estudo piloto. *J. Soc. Bras. Fonoaudiol.* 24 42–48. 10.1590/s2179-64912012000100008 22460371

[B79] Von Fox-Boyer A. (2009). *TROG-D: Test Zur Überprüfung Des Grammatikverständnisses.* Idstein: Schulz-Kirchner.

[B80] Vukovic R. K. Siegel L. S. (2006). The double-deficit hypothesis: a comprehensive analysis of the evidence. *J. Learn. Disabil.* 39 25–47. 10.1177/00222194060390010401 16512081

[B81] Wiens N. Gordon R. L. (2018). The case for treatment fidelity in active music interventions: why and how. *Ann. N. Y. Acad. Sci.* 1423 219–228. 10.1111/nyas.13639 29727027 PMC6215748

[B82] Woodruff Carr K. White-Schwoch T. (2014). Beat synchronization predicts neural speech encoding and reading readiness in preschoolers. *Proc. Natl. Acad. Sci. U.S.A.* 111 14559–14564. 10.1073/pnas.1406219111 25246562 PMC4210020

