# Supplementary material for: Auditory Stimulation Training With Technically Manipulated Musical Material in Preschool Children With Specific Language Impairments: An Explorative Study
Source: Front Psychol. 2019 Sep 4;10:2026. doi: 10.3389/fpsyg.2019.02026 (PMC6738197; doi:10.3389/fpsyg.2019.02026)
Supplement: Supplementary file 1 [file Table_1.DOCX]

**Appendix 1A**

Correlations (*r*) for the dependent variables for the ASTM group

| **Variables** | DS  (Pre/Post) | NR (Pre/Post) | RS (Pre/Post) | SP 4.000 Hz (Pre/Post) | SP 3.000 Hz (Pre/Post) | SP 2.000 Hz (Pre/Post) | PD (Pre/Post) | PDn (Pre/Post) |
| --- | --- | --- | --- | --- | --- | --- | --- | --- |
| Digit Span  (DS)  Pre | -/**.45**** | .**32***/.31 | **.34*/.55**** | -.12/.05 | .09/.31 | .12/.21 | .37/.12 | .16/.35 |
| Nonword Recall (NR)  Pre | **.32***/.24 | -/.**41^**^** | **.44**/.37^*^** | .08/-.05 | .31/.15 | .24/.13 | .23/.42 | .28/.23 |
| Recall of Sentences (RS) Pre | **.34*/.55^**^** | .44/**.35^*^** | -/.**69^**^** | .34/.28 | **.48*/.45^*^** | **.50**/.40^*^** | .50/.27 | -.04/.43 |
| Speech Perception (SP) at 4.000 Hz Pre | -.12/.20 | .08/-.06 | .34/.27 | -/**.64^**^** | **.72****/.33 | **.39***/.16 | .45/.05 | .17/.18 |
| Speech Perception (SP) at 3.000 Hz Pre | .09/.29 | .31/.10 | **.48*/.47^*^** | **.72**/.53^**^** | -/**.40^*^** | .58**/.27 | **.54***/.17 | .19/.27 |
| Speech Perception (SP) at 2.000 Hz Pre | .12/**.42^*^** | .24/.18 | **.50*/.41^*^** | **.39*/.62^**^** | **.58**/.63^**^** | -/.**54^**^** | .75**/.45 | .32/.49 |
| Phoneme Discrimination (PD) Pre | .37/.49 | .23/-.25 | .50/.37 | .45/**.53^*^** | **.54***/.50 | **.75****/- | -/**.19*** | .50/.38 |
| Phoneme Discrimination (PD_n_) Pre | .16/.09 | .28/.14 | -.04/.14 | .17/**.61**^*^ | .19/**.54^*^** | .32/.29 | .50/.36 | -/**.69^**^** |

*Note*. Diagonal = Correlations for Pre-Post measurements (T1 and T2). PD_n_ = Phoneme Discrimination with background noise. *Correlations significant at the 0.05 level (two-tailed). **Correlations significant at the 0.01 level (two-tailed).

**Appendix 1B**

Correlations (*r*) for the dependent variables for the PA group

| **Variables** | DS  (Pre/Post) | NR (Pre/Post) | RS (Pre/Post) | SP 4.000 Hz (Pre/Post) | SP 3.000 Hz (Pre/Post) | SP 2.000 Hz (Pre/Post) | PD (Pre/Post) | PDn (Pre/Post) |
| --- | --- | --- | --- | --- | --- | --- | --- | --- |
| Digit Span (DS) Pre | -/**.47*** | **.49*/.59**** | **.64**/.61**** | -.16/-.14 | .09/-.19 | .27/.14 | .34/.44 | -.46/-.28 |
| Nonword Recall (NR) Pre | **.49*/.47*** | -/.**72**** | **.62**/.67**** | .09/.05 | .39/.53 | .49/.52 | **.65****/.26 | .08/-.27 |
| Recall of Sentences (RS) Pre | **.64**/.52**** | **.62**/.55**** | -/**.92**** | .02/.07 | .17/.12 | .12/.38 | .17/.38 | .33/.08 |
| Speech Perception (SP) at 4.000 Hz Pre | -.16/-.10 | .09/.21 | .02/.04 | -/**.63*** | .48/.49 | .34/.43 | .41/**.75**** | **.61***/.54 |
| Speech Perception (SP) at 3.000 Hz Pre | .09/-.13 | .39/.04 | .17/.10 | .48/.37 | -/**.55*** | .56*/.37 | .32/**.66*** | .07/**.62*** |
| Speech Perception (SP) at 2.000 Hz Pre | .27/-.32 | .49/.35 | .38/.43 | .34/.36 | **.56***/.40 | -/**.43*** | .57/.34 | .21/.23 |
| Phoneme Discrimination (PD) Pre | .34/.25 | **.65*/.76**** | .38/.45 | .41/.15 | .32/.57 | .57/**.83**** | -/**.36*** | .57/.23 |
| Phoneme Discrimination (PD_n_) Pre | -.46/-.30 | .08/.29 | .08/.17 | .61*/.18 | .07/.33 | .21/.49 | .57/.15 | -/**.40*** |

*Note*. Diagonal = Correlations for Pre-Post measurements (T1 and T2). PD_n_ = Phoneme Discrimination with background noise. *Correlations significant at the 0.05 level (two-tailed). **Correlations significant at the 0.01 level (two-tailed).

**Appendix C**

Correlations (*r*) for the dependent variables for the control group

| **Variables** | DS  (Pre/Post) | NR (Pre/Post) | RS (Pre/Post) | SP 4.000 Hz (Pre/Post) | SP 3.000 Hz (Pre/Post) | SP 2.000 Hz (Pre/Post) | PD (Pre/Post) | PD_n_ (Pre/Post) |
| --- | --- | --- | --- | --- | --- | --- | --- | --- |
| Digit Span (DS) Pre | -/**.49**** | .26/**.35*** | **.53**/.39*** | -.03/-.11 | .03/.12 | .39/.30 | .35/**.49*** | **.47***/.14 |
| Nonword Recall (NR) Pre | .26/.28 | -/**.68**** | **.43****/.26 | -.13/-.04 | .10/**.42*** | .01/.39 | .26/.35 | .40/.13 |
| Recall of Sentences (RS) Pre | **.53**/.47**** | **.43**/.64**** | -/**.71**** | .28/.20 | .40/.30 | **.43***/.35 | **.57**/.57**** | **.57****/.31 |
| Speech Perception (SP) at 4.000 Hz Pre | -.03/.21 | .13/.13 | .28/.24 | -/**.56**** | **.48***/12 | .26/.15 | -.07/.18 | .26/.41 |
| Speech Perception (SP) at 3.000 Hz Pre | .03/-.10 | .10/.20 | .40/**.50*** | **.48***/.16 | -/**.45*** | **.60****/.27 | **.50***/.40 | **.59**/.68**** |
| Speech Perception (SP) at 2.000 Hz Pre | .39/.08 | .01/.21 | **.43***/.33 | .26/.23 | **.60****/.35 | -/**.44*** | .37/**.47*** | **.57**/.50*** |
| Phoneme Discrimination (PD) Pre | .35/.36 | .26/**.41*** | **.57**/.49*** | -.07/-.21 | **.50***/-.07 | .37/-.01 | -/**.66**** | **.74****/.40 |
| Phoneme Discrimination (PD_n_) Pre | **.47***/.38 | .40/**.50*** | .**57**/.60**** | .26/-.01 | **.59****/.13 | **.57****/.10 | **.74**/.52*** | -/**.59**** |

*Note*. Diagonal = Correlations for Pre-Post measurements for dependent variables. PD_n_ = Phoneme Discrimination with background noise. *Correlations significant at the 0.05 level (two-tailed). **Correlations significant at the 0.01 level (two-tailed).
