# Supplementary material for: Auditory Stimulation Training With Technically Manipulated Musical Material in Preschool Children With Specific Language Impairments: An Explorative Study
Source: Front Psychol. 2019 Sep 4;10:2026. doi: 10.3389/fpsyg.2019.02026 (PMC6738197; doi:10.3389/fpsyg.2019.02026)
Supplement: Supplementary file 2 [file Table_2.DOCX]

Appendix 2


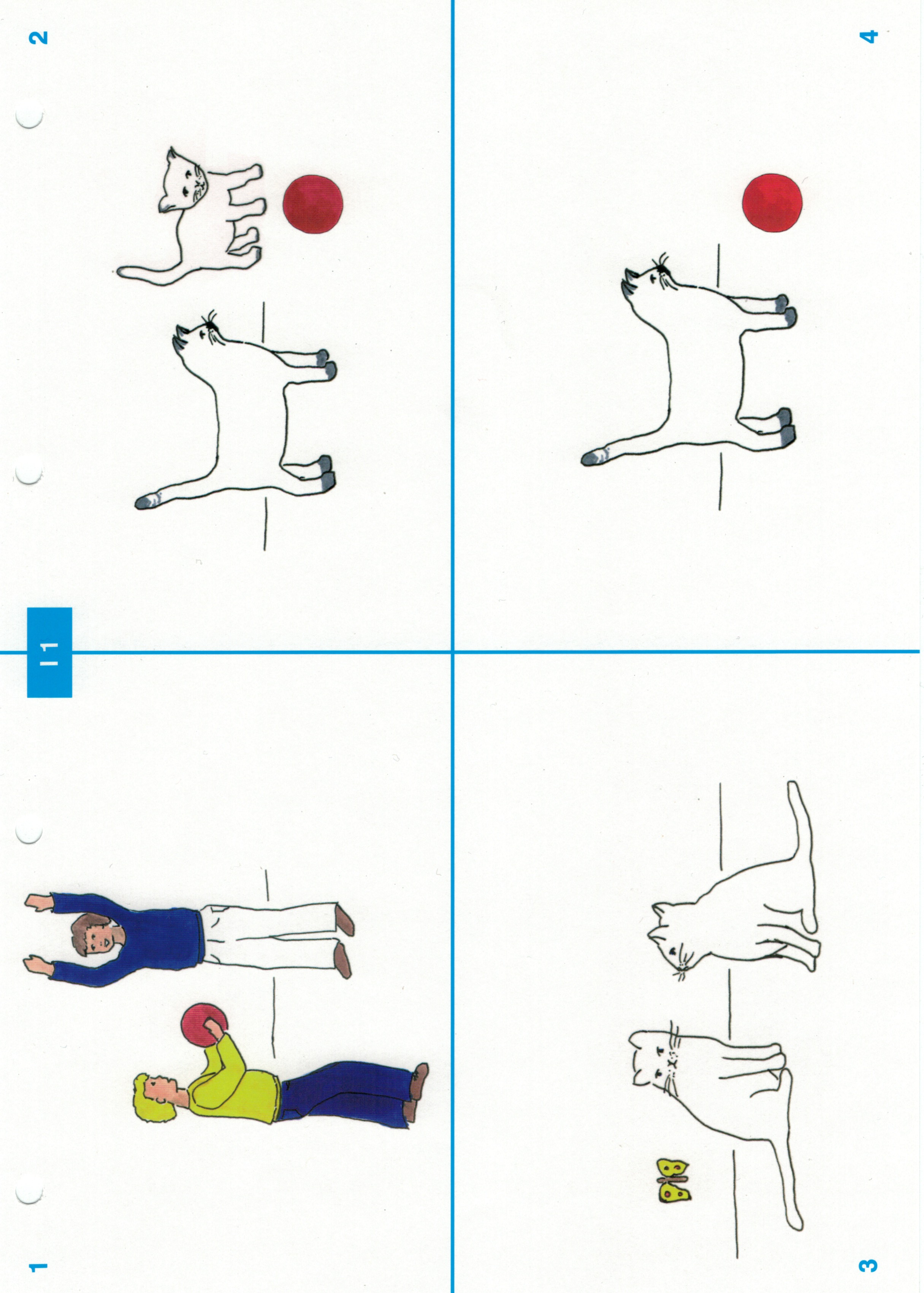


**Example of the Reception of Grammar Test (TROG-D).** The participants were asked to select one of the four images that match to a readout sentence. In this example, the corresponding image to the sentence “The cats were looking at the ball” is image 2.
