# Supplementary material for: Auditory Stimulation Training With Technically Manipulated Musical Material in Preschool Children With Specific Language Impairments: An Explorative Study
Source: Front Psychol. 2019 Sep 4;10:2026. doi: 10.3389/fpsyg.2019.02026 (PMC6738197; doi:10.3389/fpsyg.2019.02026)
Supplement: Supplementary file 3 [file Table_3.docx]

Appendix 3: Stimuli for the Phoneme Discrimination Test (PD)

| PD without background noise | | PD with background noise | |
| --- | --- | --- | --- |
| 1. | AFI | 1. | OTI |
| 2. | IDA | 2. | AWE |
| 3. | EBU | 3. | IPA |
| 4. | AGO | 4. | UKA |
| 5. | OTE | 5. | AFO |
| 6. | AKO | 6. | EPA |
| 7. | IBA | 7. | EWU |
| 8. | EGI | 8. | ADI |
| 9. | OTI | 9. | AFI |
| 10. | AWE | 10. | IDA |
| 11. | IPA | 11. | EBU |
| 12. | UKA | 12. | AGO |
| 13. | AFO | 13. | OTE |
| 14. | EPA | 14. | AKO |
| 15. | EWU | 15. | IBA |
| 16. | ADI | 16. | EGI |
| 17. | OTE | 17. | EWO |
| 18. | EFO | 18. | IGA |
| 19. | ADI | 19. | EDU |
| 20. | IKE | 20. | IWE |
| 21. | EBI | 21. | EBU |
| 22. | IGU | 22. | OTE |
| 23. | EPO | 23. | IFA |
| 24. | AKE | 24. | IPU |
| 25. | EWO | 25. | OTE |
| 26. | IGA | 26. | EFO |
| 27. | EDU | 27. | ADI |
| 28. | IWE | 28. | IKE |
| 29. | EBU | 29. | EBI |
| 30. | OTE | 30. | IGU |
| 31. | IFA | 31. | EPO |
| 32. | IPU | 32. | AKE |
